# Ebola Viral Disease Outbreak — West Africa, 2014

**Published:** 2014-06-27

**Authors:** Meredith G. Dixon, Ilana J. Schafer

**Affiliations:** 1 EIS officer, CDC; 2 Viral Special Pathogens Branch, National Center for Emerging and Zoonotic Infectious Diseases, CDC

On March 21, 2014, the Guinea Ministry of Health reported the outbreak of an illness characterized by fever, severe diarrhea, vomiting, and a high case-fatality rate (59%) among 49 persons (1). Specimens from 15 of 20 persons tested at Institut Pasteur in Lyon, France, were positive for an Ebola virus by polymerase chain reaction (2). Viral sequencing identified Ebola virus (species *Zaïre ebolavirus*), one of five viruses in the genus *Ebolavirus*, as the cause (2). Cases of Ebola viral disease (EVD) were initially reported in three southeastern districts (Gueckedou, Macenta, and Kissidougou) of Guinea and in the capital city of Conakry. By March 30, cases had been reported in Foya district in neighboring Liberia (1), and in May, the first cases identified in Sierra Leone were reported. As of June 18, the outbreak was the largest EVD outbreak ever documented, with a combined total of 528 cases (including laboratory-confirmed, probable, and suspected cases) and 337 deaths (case-fatality rate = 64%) reported in the three countries. The largest previous outbreak occurred in Uganda during 2000–2001, when 425 cases were reported with 224 deaths (case-fatality rate = 53%) (3). The current outbreak also represents the first outbreak of EVD in West Africa (a single case caused by Taï Forest virus was reported in Côte d’Ivoire in 1994 [3]) and marks the first time that Ebola virus transmission has been reported in a capital city.

## Characteristics of EVD

EVD is characterized by the sudden onset of fever and malaise, accompanied by other nonspecific signs and symptoms such as myalgia, headache, vomiting, and diarrhea. Among EVD patients, 30%–50% experience hemorrhagic symptoms (4). In severe and fatal forms, multiorgan dysfunction, including hepatic damage, renal failure, and central nervous system involvement occur, leading to shock and death. The first two *Ebolavirus* species were initially recognized in 1976 during simultaneous outbreaks in Sudan (*Sudan ebolavirus*) and Zaïre (now Democratic Republic of the Congo) (*Zaïre ebolavirus*) (5). Since 1976, there have been more than 20 EVD outbreaks across Central Africa, with the majority caused by Ebola virus (species *Zaïre ebolavirus*), which historically has demonstrated the highest case-fatality rate (up to 90%) (3).

The wildlife reservoir has not been definitively ascertained; however, evidence supports fruit bats as one reservoir (6). The virus initially is spread to the human population after contact with infected wildlife and is then spread person-to-person through direct contact with body fluids such as, but not limited to, blood, urine, sweat, semen, and breast milk. The incubation period is 2–21 days. Patients can transmit the virus while febrile and through later stages of disease, as well as postmortem, when persons contact the body during funeral preparations. Additionally, the virus has been isolated in semen for as many as 61 days after illness onset.

Diagnosis is made most commonly through detection of Ebola virus RNA or Ebola virus antibodies in blood (5). Testing in this outbreak is being performed by Institut Pasteur, the European Mobile Laboratory, and CDC in Guinea; by the Kenema Government Hospital Viral Hemorrhagic Fever Laboratory in Sierra Leone; and by the Liberia Institute of Biomedical Research. Patient care is supportive; there is no approved treatment known to be effective against Ebola virus. Clinical support consists of aggressive volume and electrolyte management, oral and intravenous nutrition, and medications to control fever and gastrointestinal distress, as well as to treat pain, anxiety, and agitation (4,5). Diagnosis and treatment of concomitant infections and superinfections, including malaria and typhoid, also are important aspects of patient care (4).

Keys to controlling EVD outbreaks include 1) active case identification and isolation of patients from the community to prevent continued virus spread; 2) identifying contacts of ill or deceased persons and tracking the contacts daily for the entire incubation period of 21 days; 3) investigation of retrospective and current cases to document all historic and ongoing chains of virus transmission; 4) identifying deaths in the community and using safe burial practices; and 5) daily reporting of cases (4,7,8). Education of health-care workers regarding safe infection-control practices, including appropriate use of personal protective equipment, is essential to protect them and their patients because health-care–associated transmission has played a part in transmission during previous outbreaks (4,9).

## Efforts to Control the Current Outbreak

To implement prevention and control measures in both Guinea and Liberia, ministries of health with assistance from Médecins Sans Frontières, the World Health Organization, and others, put in place Ebola treatment centers to provide better patient care and interrupt virus transmission. Teams from CDC traveled to Guinea and Liberia at the end of March as part of a response by the Global Outbreak Alert and Response Network to assist the respective ministries of health in characterizing and controlling the outbreak through collection of case reports, interviewing of patients and family members, coordination of contact tracing, and consolidation of data into centralized databases. Cases are categorized into one of three case definitions: suspected (alive or dead person with fever and at least three additional symptoms, or fever and a history of contact with a person with hemorrhagic fever or a dead or sick animal, or unexplained bleeding); probable (meets the suspected case definition and has an epidemiologic link to a confirmed or probable case); confirmed (suspected or probable case that also has laboratory confirmation).*

**FIGURE 1:**
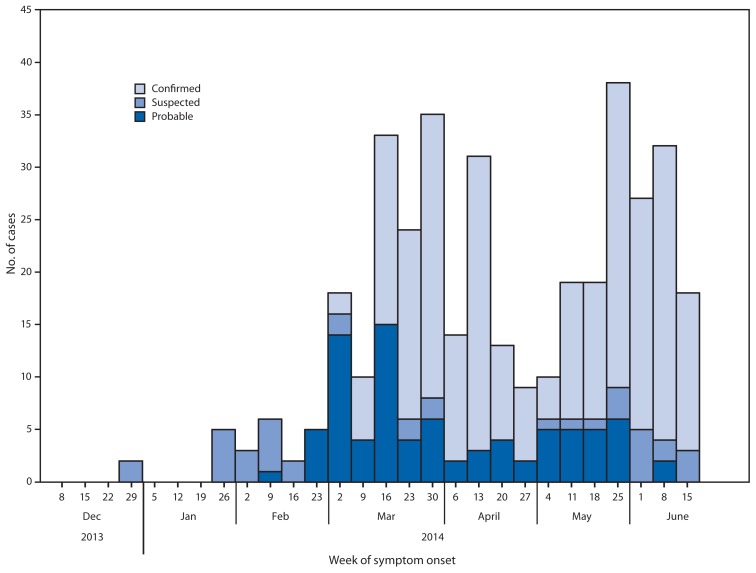
Number of cases of Ebola viral disease (n = 398^*^), by week of symptom onset — Guinea, 2014 * Cases reported as of June 18, 2014.

In late April, it appeared that the outbreak was slowing when Liberia did not report new cases for several weeks after April 9, and the number of new reported cases in Guinea decreased to nine for the week of April 27 (Figure 1). Since then, however, the EVD outbreak has resurged, with neighboring Sierra Leone reporting its first laboratory-confirmed case on May 24, Liberia reporting a new case on May 29 that originated in Sierra Leone, and Guinea reporting a new high of 38 cases for the week of May 25.

As of June 18, the total EVD case count reported for all three countries combined was 528, including 364 laboratory-confirmed, 99 probable, and 65 suspected cases, with 337 deaths (case-fatality rate = 64%). Guinea had reported 398 cases (254 laboratory-confirmed, 88 probable, and 56 suspected) with 264 deaths (case-fatality rate = 66%) across nine districts (Figure 1). Sierra Leone had reported 97 cases (92 laboratory-confirmed, three probable, and two suspected) with 49 deaths (case-fatality rate = 51%) across five districts and the capital, Freetown. Liberia had reported 33 cases (18 confirmed, eight probable, and seven suspected) with 24 deaths (case-fatality rate = 73%) across four districts.

**FIGURE 2:**
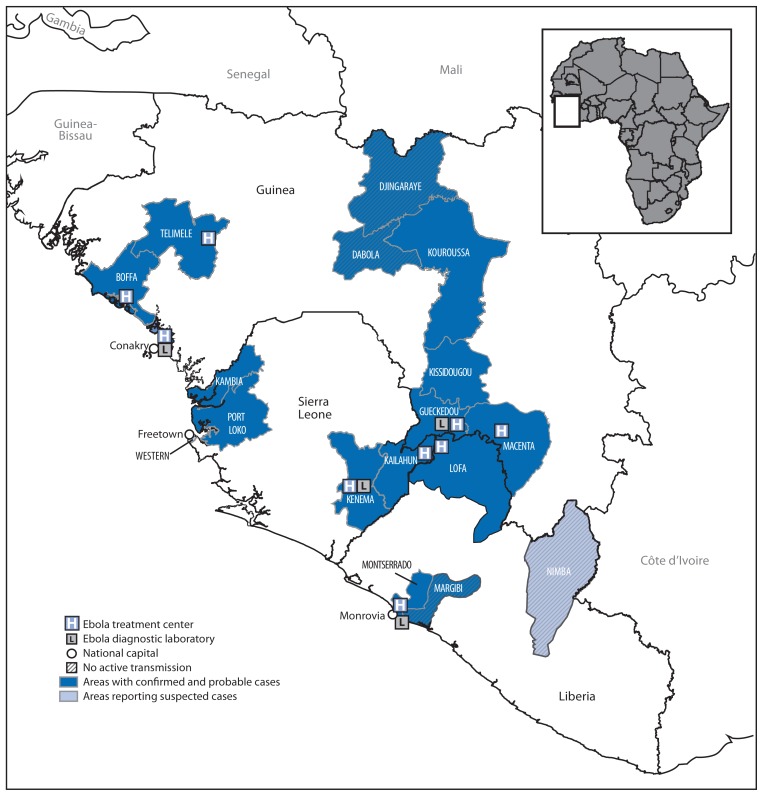
Location of cases of Ebola viral disease* — West Africa, 2014 * Cases reported as of June 18, 2014.

Major challenges faced by all partners in the efforts to control the outbreak include its wide geographic spread (Figure 2), weak health-care infrastructures, and community mistrust and resistance (10). Retrospective case investigation has indicated that the first case of EVD might have occurred as early as December 2013 (Figure 1) (2). To control the outbreak, additional strategies such as involving community leaders in response efforts are needed to alleviate concerns of hesitant and fearful populations so that health-care workers can care for patients in treatment centers and thorough contact tracing can be performed. Enhancing communication across borders with respect to disease surveillance will assist in the control and prevention of more cases in this EVD outbreak.

In June 2014, the World Health Organization, via the Global Outbreak Alert and Response Network, requested additional support from CDC and other partners, necessitating the deployment of additional staff members to Guinea and Sierra Leone to further coordinate efforts aimed at halting and preventing virus transmission. Persistence of the outbreak necessitates high-level, regional and international coordination to bolster response efforts among involved and neighboring nations and other response partners in order to expeditiously end this outbreak.

## Footnotes

* Case definitions were modified from those available at http://who.int/csr/resources/publications/ebola/ebola-case-definition-contact-en.pdf.
